# The Pathogenic Aβ43 Is Enriched in Familial and Sporadic Alzheimer Disease

**DOI:** 10.1371/journal.pone.0055847

**Published:** 2013-02-11

**Authors:** Anna Sandebring, Hedvig Welander, Bengt Winblad, Caroline Graff, Lars O. Tjernberg

**Affiliations:** 1 Department of Neurobiology, Care Sciences and Society, Karolinska Institutet, KI-Alzheimer’s Disease Research center (KI-ADRC), Huddinge, Sweden; 2 Department of Public Health/Molecular Geriatrics, Rudbeck Laboratory, Uppsala University, Uppsala, Sweden; University of Florida, United States of America

## Abstract

The amyloid-cascade hypothesis posits that the role of amyloid β-peptide (Aβ) in Alzheimer disease (AD) involves polymerization into structures that eventually are deposited as amyloid plaques. During this process, neurotoxic oligomers are formed that induce synaptic loss and neuronal death. Several different isoforms of Aβ are produced, of which the 40 and 42 residue variants (Aβ40 and Aβ42) are the most common. Aβ42 has a strong tendency to form neurotoxic aggregates and is involved in AD pathogenesis. Longer Aβ isoforms, like the less studied Aβ43, are gaining attention for their higher propensity to aggregate into neurotoxic oligomers. To further investigate Aβ43 in AD, we conducted a quantitative study on Aβ43 levels in human brain. We homogenized human brain tissue and prepared fractions of various solubility; tris buffered saline (TBS), sodium dodecyl sulfate (SDS) and formic acid (FA). Levels of Aβ43, as well as Aβ40 and Aβ42, were quantified using ELISA. We compared quantitative data showing Aβ levels in occipital and frontal cortex from sporadic (SAD) and familial (FAD) AD cases, as well as non-demented (ND) controls. Results showed Aβ43 present in each fraction from the SAD and FAD cases, while its level was lower than the detection limit in the majority of the ND-cases. Aβ42 and Aβ43 were enriched in the less soluble fractions (SDS and FA) of SAD and FAD cases in both occipital and frontal cortex. Thus, although the total levels of Aβ43 in human brain are low compared to Aβ40 and Aβ42, we suggest that Aβ43 could initiate the formation of oligomers and amyloid plaques and thereby be crucial to AD pathogenesis.

## Introduction

Alzheimer disease (AD) is a neurodegenerative disorder and the most common form of dementia. Toxic oligomeric species of the amyloid β-peptide (Aβ) in AD induce synaptic degeneration and neuronal death in the affected brains. The “amyloid cascade hypothesis” posits that accumulation of Aβ in the brain is the principal cause for AD pathogenesis [1], [2]. An imbalance in the production and clearance of Aβ may lead to Aβ oligomerization, fibril formation, and eventual deposition of Aβ into amyloid plaques. Initially, it was believed that the insoluble fibrillar deposits of Aβ found in plaques were the key mediators of AD. However, the levels of soluble Aβ oligomers more strongly correlate with disease and thus are today considered to be the most toxic species [1].

Amyloid precursor protein (APP) is sequentially proteolytically processed by β-secretase and γ-secretase to create Aβ, which is eventually found deposited in AD brains as amyloid plaques and in cerebral blood vessel walls [3], [4]. β-Secretase cleaves APP [5] at the intralumenal/extracellular side, generating soluble βAPP and a 99-residue membrane bound fragment that is the immediate substrate of γ-secretase. γ-Secretase is an aspartyl transmembrane protease complex containing four proteins: presenilin (PS), nicastrin, anterior pharynx defective-1 (Aph-1), and presenilin enhancer-2 (Pen-2) [6]. γ-Secretase sequentially cleaves APP at different locations in the transmembrane domain. The first cut, ε-cleavage, occurs in the membrane near the cytoplasm and is followed by ζ- and γ-cleavages in the middle of the transmembrane domain [7], [8], [9], [10], resulting in the release of Aβ species of different lengths [11], [12]. Previous studies show compelling evidence that the γ-secretase complex produces Aβ in a sequential manner through tri- or tetra-peptide cleavage of the APP-CTF, generating species of steadily decreasing length and hydrophobicity [11], [13]. Either of two separate pathways may be followed depending on the initial ε-cleavage site: Aβ49→Aβ46→Aβ43→Aβ40 or Aβ48→Aβ45→Aβ42→Aβ38 [12]. Of these species, Aβ40 and Aβ42 are the most abundant [14]. Aβ40 is produced at higher levels and is predominantly observed in cerebral blood vessels [15], [16], cerebrospinal fluid (CSF) and plasma. Aβ42 is more hydrophobic and, thereby, more prone to polymerize into neurotoxic aggregates. Accordingly, Aβ42 is of particular importance in AD pathogenesis [1], [16], [17], [18], [19]. Variants longer than Aβ42 are even more hydrophobic and polymerize faster. Such species, including Aβ43, Aβ45, Aβ48, Aβ49 and Aβ50, have been identified in cell lines [11], [20] and transgenic mice [21], [22], [23] and reported in a few studies in human brain [24], [25]. Furthermore, in two earlier quantitative studies deposits of Aβ43 were more frequent than Aβ40 in different brain regions obtained from both sporadic and familial AD (FAD) [26], [27].

The cause of early-onset FAD is mutations in genes encoding APP [28], presenilin 1 (*PSEN 1*) [29] or presenilin 2 (*PSEN 2*) [30]. These mutations lead to increased production of Aβ42 or to increased ratio of Aβ42/Aβ40 [31], [32], further implicating the importance of Aβ42 in AD pathogenesis. Analysis of FAD cases caused by *PSEN1* mutations show that a decreased production of shorter Aβ species shifts the generation of Aβ toward greater Aβ43/Aβ40 and Aβ42/Aβ38 ratios [33]. Results from the same study showed that FAD-*APP* mutations appear to shift the initial ε-cleavage towards the Aβ48 sequence. That study and a second recent study of different FAD mutations, have each concluded that elevated ratios of Aβ42/Aβ38 as well as Aβ43/Aβ40 are important factors for AD pathology [33], [34].

Trace amounts of Aβ42 and Aβ43 are critical for amyloid plaque formation *in vivo*, and can seed Aβ40 polymerization [17], [35], [36]. For a recent study Saito and colleagues [34] created a knock-in mouse that overproduces Aβ43. Subsequently, they demonstrated that Aβ43 was abundant *in vivo*, exhibited a greater propensity to aggregate, provoked Aβ42 polymerization, and was more neurotoxic than Aβ42. Recently, Aβ43 was identified and quantified in CSF from human AD/mild cognitive impairment (MCI) patients and control subjects using a commercial Aβ43 enzyme-linked immunosorbent assay (ELISA) [37]. Concentrations in CSF of both Aβ42 and Aβ43 were significantly lower in the patients compared with control subjects indicating an accumulation of these peptides in the brain. Moreover, Aβ43 has been identified in dog CSF in a study using peptide adsorption – liquid chromatography – tandem mass spectrometry [38]. Although these recent publications have shed more light on Aβ43, more studies are warranted to learn more about the impact of Aβ43 on AD pathogenesis.

Therefore, in the present study, we analyzed and quantified Aβ43 in human brain in tris-buffered saline (TBS), sodium dodecyl sulfate (SDS) and formic acid (FA) soluble fractions from inferior frontal and secondary occipital cortex, from SAD and FAD, as well as in non-demented (ND) cases. The prefrontal cortex is typically associated with cognitive tasks involving executive functions, and the right side inferior frontal gyrus is associated to response inhibition and task-set switching [39]. The secondary occipital gyrus receives and processes input from the primary visual cortex. Importantly, both regional targets of analysis in this study are affected in AD [40], [41]. In summary, our data show that Aβ43 accumulates in SAD and FAD brains, but not in ND cases. We further show that Aβ43 is enriched in the less soluble fractions in a manner similar to Aβ42.

## Materials and Methods

### Human Subjects

**Figure 1:**
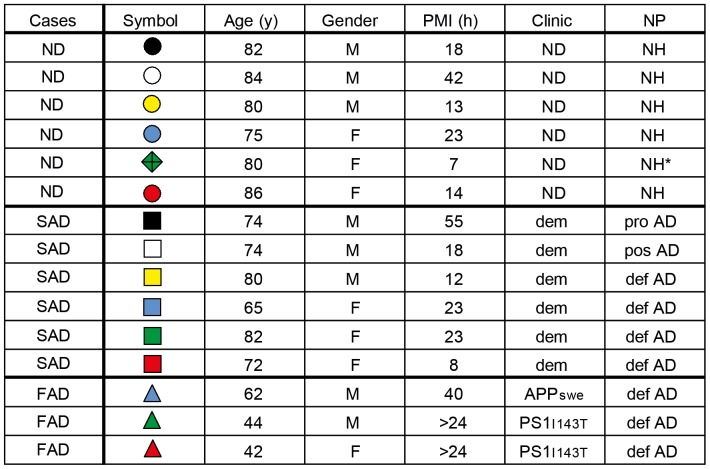
Study Subject Data. ND = non-demented; SAD = sporadic Alzheimer disease; FAD = familial Alzheimer disease; y = years; M = male; F = female; PMI = postmortem interval; h = hours; Clinic = clinical neurological diagnosis; dem = demented; APPswe = Swedish mutation in *APP*; PS1I143T = I143T mutation in *PSEN1*; NP = neuropathological diagnosis; NH = neurologically healthy; pro AD = probable AD; pos AD = possible AD; def AD = definite AD. NH* = case excluded from grouped analysis.

Human brain specimens were obtained from the Brain bank at Karolinska Institutet (2011/962-31/1). Samples from six cognitively healthy non-demented (ND) individuals (three males and three females) were included. Nine cases with clinical dementia were included, six that had subsequently been neuropathologically diagnosed with SAD and three FAD cases (one *APP* Swedish mutation carrier and two *PSEN1* Ile143Thr mutation carriers). The postmortem intervals (PMI), neuropathological (NP) and clinical diagnose, age and gender distribution by diagnostic group, including ND, SAD and FAD are presented in **Figure 1**.

### Preparation of Human Samples for ELISA Analysis

One gram of frozen material each from inferior frontal cortex and secondary occipital cortex were homogenized in 12 ml 1 × TBS (50 mM Tris-HCl, 0.15 M NaCl, pH 7.4), using a dounce homogenizer for 25 strokes at 1500 rpm. Homogenization was performed at 4°C in the presence of a protease inhibitor cocktail (Complete protease inhibitor from Roche, Basel, Switzerland). Brain homogenate (3 ml each, corresponding to 0.25 g of wet tissue) was centrifuged at 175,000 × g for 20 minutes. The supernatant (TBS soluble fraction) was collected and the pellet dissolved in 3 ml 1% sodium dodecyl sulfate (SDS) in 1 × TBS, then diluted 10 times (300 µl +2700 µl) in 1% SDS and centrifuged at 175,000 × g for 20 minutes at room temperature. The supernatant was collected and the pellet was homogenized in 1% SDS followed by centrifugation. This step was repeated until four SDS soluble fractions were obtained. The remaining pellet of SDS insoluble material was immediately dissolved in 80% formic acid (FA) then vortexed for 30 seconds followed by 20 minutes sonication at 20°C in a water bath. Samples were centrifuged at 25,000 × g for 10 minutes and the supernatant was collected for further analysis. All samples were stored at −20°C. Immediately before ELISA measurements, FA soluble fractions were neutralized in 2 M Tris-HCl, pH 9.0 and mixed with 5× Radio-immunoprecipitation assay (RIPA) buffer (750 mM NaCl/5.0% NP-40/2.5% sodium deoxycholate/0.5% SDS/250 mM Tris–HCl, pH 8.0), to avoid peptide aggregation.

### Chemicals

Trizma base, FA, SDS, NaCl, NP-40 and sodium deoxycholate were obtained from Sigma Aldrich (St Louis, MO, USA).

### Enzyme Linked Immunosorbent Assay (ELISA)

Aβ concentrations in TBS, SDS and FA fractions were measured in duplicate by human/rat β amyloid (40) ELISA kit WakoII (Wako Chemicals GmbH, Neuss, Germany), human/rat β amyloid (42) ELISA kit Wako High-Sensitive (Wako Chemicals GmbH, Neuss, Germany) and amyloid-β (1–43) (FL) ELISA (Immuno-biological Laboratories, Hamburg, Germany). The protocol was according to manufacturer’s instructions except for quantification of the antibody binding, which was instead obtained by incubation with 50 µM Amplex UltraRed reagent (Invitrogen, Täby, Sweden) for 30 minutes, followed by detection of fluorescent signal, using a 544 nm excitation filter and 590 nm emission filter in a microplate reader (TECAN, Männedorf, Switzerland). Each sample was measured in duplicate.

### Protein Determination

Protein concentrations were determined using Pierce BCA kit (Thermo Scientific, Rockford, IL, USA) according to manufacturer’s instructions.

### Statistical Analysis

Data are presented in scatter plots, in which each symbol represents one case as listed in **Figure 1** and group means are indicated with horizontal lines. Between group analyses of variables were performed by the Mann-Whitney U test (*<0.05, **<0.01; #<0.05, ##<0.01).

## Results

This study compared three different Aβ species - Aβ40, Aβ42 and Aβ43, in two different cortical regions of the human brain. Homogenates from ND, SAD and FAD cases were prepared from frontal and occipital cortex and subsequently separated into three fractions of different solubility: soluble (TBS), soluble in 1% SDS (SDS), and soluble in FA (FA). The Aβ levels for each individual case are represented as unique colored symbols all through the figures. The study subject data can be found in **Fig. 1**.

### ELISA Quantifications

**Figure 2:**
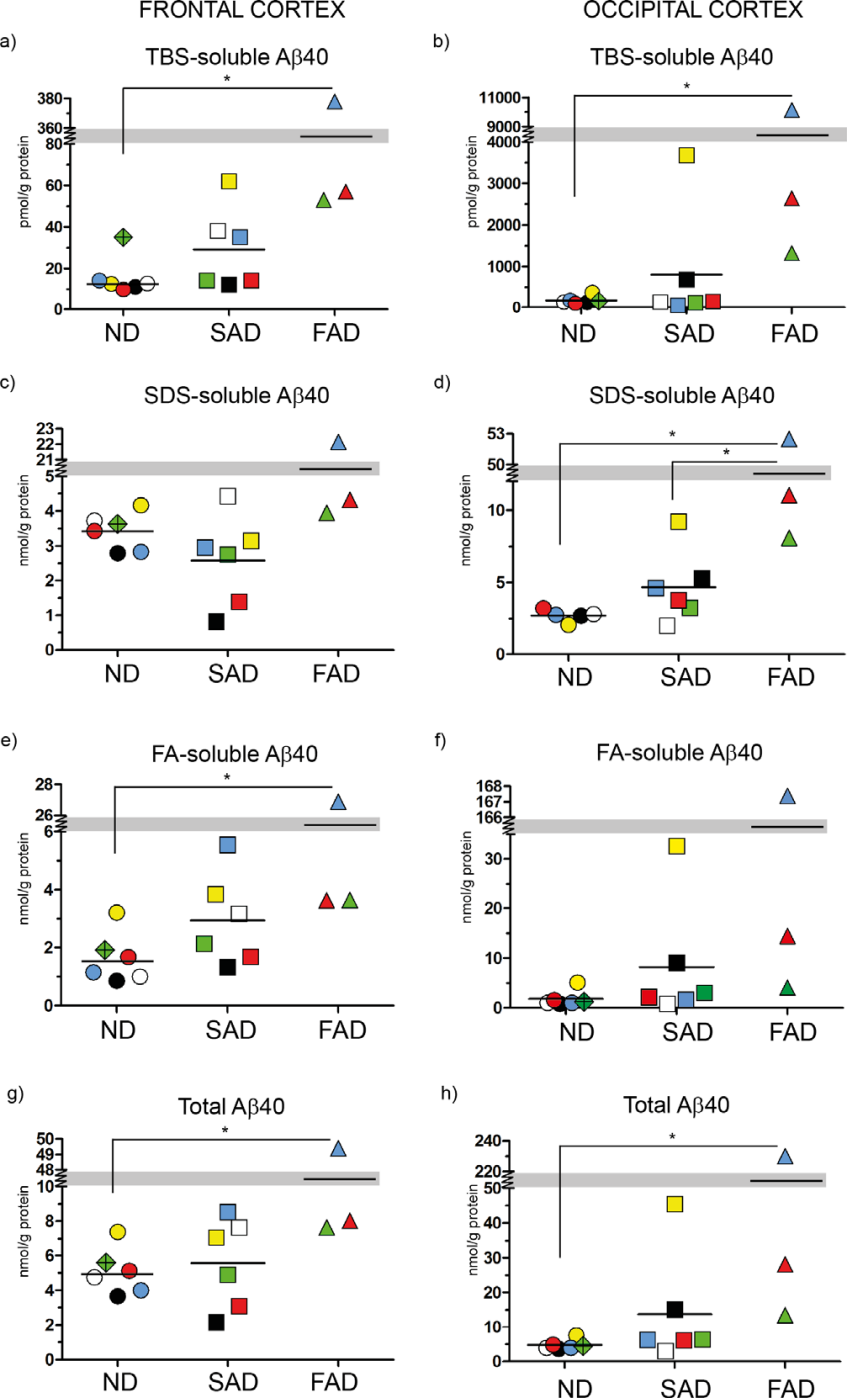
ELISA scatter plots of Aβ40. Fractions of human brain homogenates from non-demented (ND), sporadic Alzheimer disease (SAD) and familial Alzheimer disease (FAD) were analyzed with an Aβ40-specific ELISA. Colored symbols each represents one case as listed in **Figure 1** and horizontal lines indicate the mean value of each group. Data is expressed as nmol or pmol/g of protein. **a**) TBS-soluble Aβ40 in frontal cortex; **b**) TBS-soluble Aβ40 in occipital cortex; **c**) SDS-soluble Aβ40 in frontal cortex; **d**) SDS-soluble Aβ40 in occipital cortex; **e**) FA-soluble Aβ40 in frontal cortex; **f**) FA-soluble Aβ40 in occipital cortex; **g**) Total Aβ40 (TBS+SDS+FA) in frontal cortex; **h**) Total Aβ40 (TBS+SDS+FA) in occipital cortex *<0.05; **<0.01.

Immunoassays for Aβ40, Aβ42 and Aβ43 were performed for each ND, SAD and FAD case and the amount of Aβ was calculated and expressed in nmol or pmol/g protein as shown in **Fig. 2** **,** **3** and **4** (mean group concentrations are listed in **Table S1**). One ND case was excluded from statistical analysis due to high Aβ levels and diffuse plaques, which indicated pre-symptomatic AD. However, this case is represented throughout the graphs as a green diamond.

**Figure 3:**
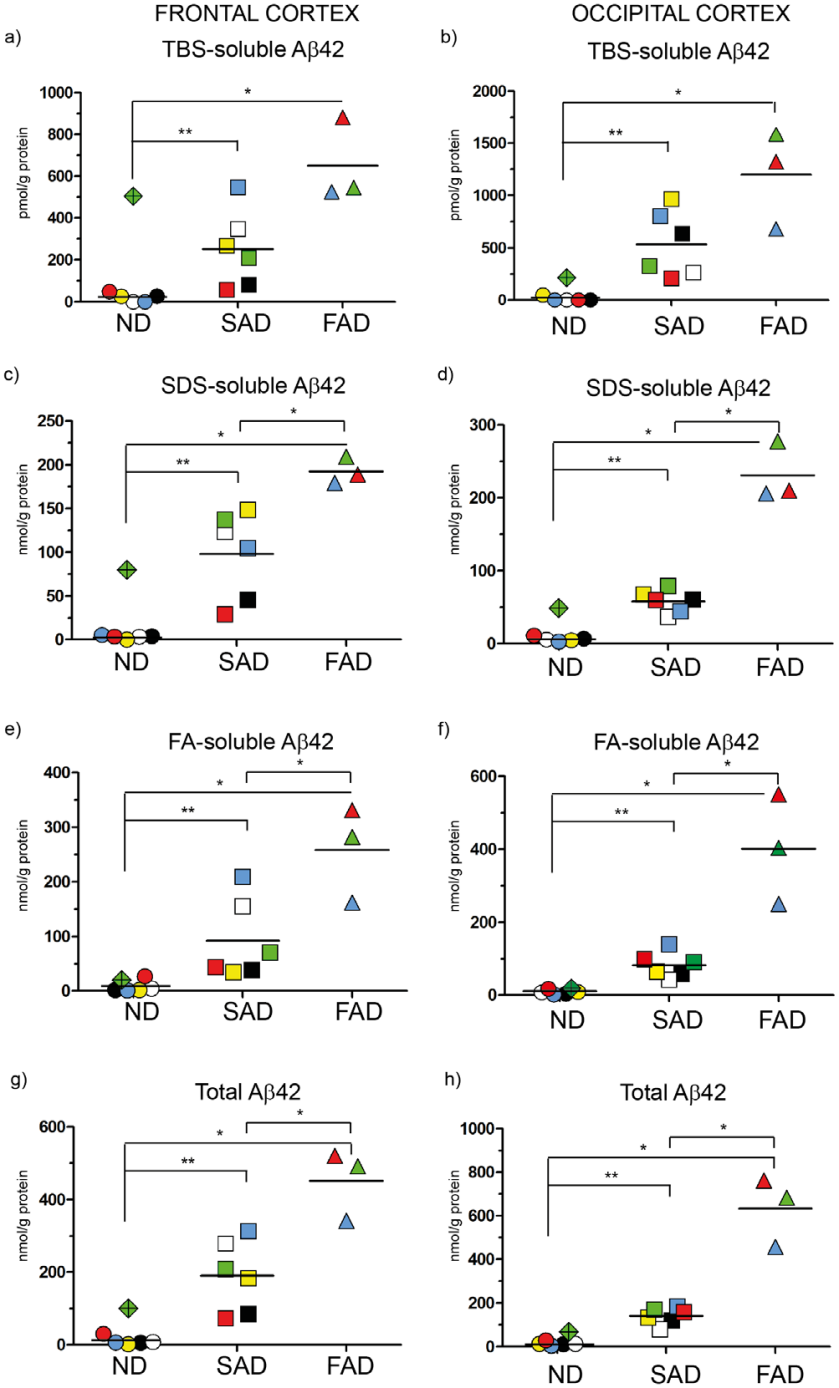
ELISA scatter plots of Aβ42. Fractions of human brain homogenates from non-demented (ND), sporadic Alzheimer disease (SAD) and familial Alzheimer disease (FAD) were analyzed with an Aβ42-specific ELISA. Colored symbols each represents one case as listed in **Figure 1** and horizontal lines indicate the mean value of each group. Data is expressed as nmol or pmol/g of protein. **a**) TBS-soluble Aβ42 in frontal cortex; **b**) TBS-soluble Aβ42 in occipital cortex; **c**) SDS-soluble Aβ42 in frontal cortex; **d**) SDS-soluble Aβ42 in occipital cortex; **e**) FA-soluble Aβ42 in frontal cortex; **f**) FA-soluble Aβ42 in occipital cortex; **g**) Total Aβ42 (TBS+SDS+FA) in frontal cortex; **h**) Total Aβ42 (TBS+SDS+FA) in occipital cortex *<0.05; **<0.01.

**Figure 4:**
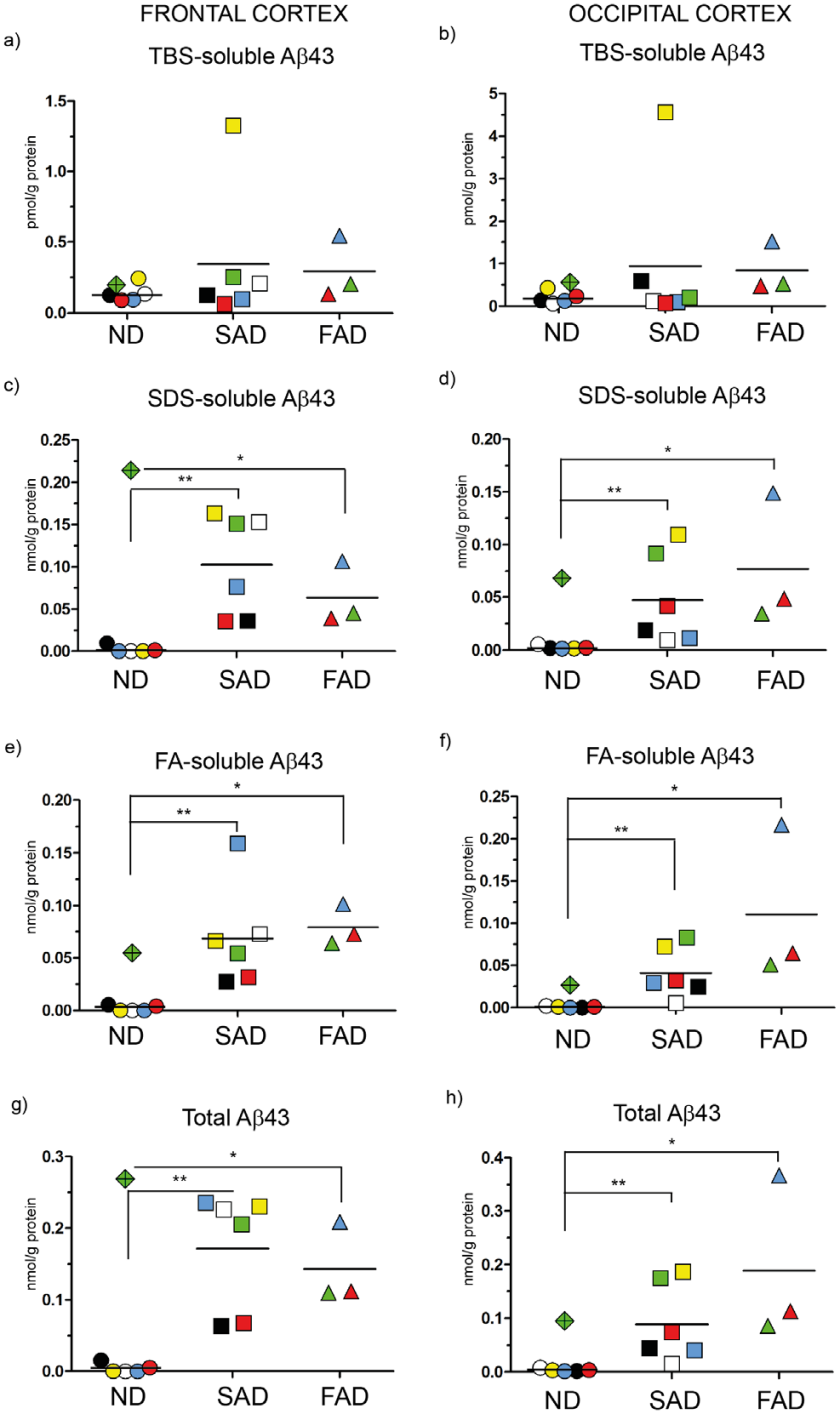
ELISA scatter plots of Aβ43. Fractions of human brain homogenates from non-demented (ND), sporadic Alzheimer disease (SAD) and familial Alzheimer disease (FAD) were analyzed with an Aβ43-specific ELISA. Colored symbols each represents one case as listed in **Figure 1** and horizontal lines indicate the mean value of each group. Data is expressed as nmol or pmol/g of protein. **a**) TBS-soluble Aβ43 in frontal cortex; **b**) TBS-soluble Aβ43 in occipital cortex; **c**) SDS-soluble Aβ43 in frontal cortex; **d**) SDS-soluble Aβ43 in occipital cortex; **e**) FA-soluble Aβ43 in frontal cortex; **f**) FA-soluble Aβ43 in occipital cortex; **g**) Total Aβ43 (TBS+SDS+FA) in frontal cortex; **h**) Total Aβ43 (TBS+SDS+FA) in occipital cortex *<0.05; **<0.01.

### Analysis of Aβ40 Levels

Although Aβ40 is highly produced, it does not seem to exhibit neurotoxic effects in AD. In this study, analysis of Aβ40 levels revealed that there were no significant differences between ND and SAD cases in any of the fractions (**Fig. 2**), implying that Aβ40 is a non-pathogenic Aβ peptide. However, one FAD case, the *APPswe* mutant (blue triangle), exhibited extremely high accumulation of Aβ40 in the brain, which indeed had a strong impact on the FAD group mean. Thus, although the two other FAD cases (*PSEN1* mutants) had moderate Aβ40 levels in all fractions, the mean for the FAD group was significantly higher than the ND group mean in frontal cortex TBS-soluble fraction (**Fig. 2a**), FA-soluble fraction (**Fig. 2e**) and in the total Aβ fraction (**Fig. 2g**); but not in the SDS-soluble fraction (**Fig. 2c**). This finding supports the hypothesis that this particular mutation, in contrast to most *PSEN* mutations, leads to a general increase in Aβ production. In occipital cortex, Aβ40 was, both in total and in the each of the TBS and SDS fractions, significantly higher in the FAD cases compared to the ND group (**Fig. 2b, 2d and 2h**) but not higher in the FA-soluble fraction (**Fig. 2f**). Except for the occipital SDS-soluble fraction (**Fig. 2d**), no significant differences were obtained between the SAD and FAD groups means for any fractions analyzed (**Fig. 2b, 2f and 2h**).

In summary, no significant differences in Aβ40 levels were obtained between ND and SAD, confirming that this species holds less importance for AD. The FAD case carrying the *APPswe* mutation differed fundamentally from all other cases, exhibiting extremely high Aβ40 levels in all fractions analyzed. Thus, when highly expressed, even the relatively harmless Aβ40 could contribute to the pathology of the disease.

### Analysis of Aβ42 Levels

Next, we wanted to determine the presence of pathogenic Aβ species in the studied cohort, and we therefore measured the concentration of Aβ42 in all fractions (**Fig. 3**). In this part of the study, we expected clear differences between AD and ND brain, since Aβ42 is neurotoxic and is believed to play a key role in AD pathogenesis [3], [15]. Indeed, in contrast to Aβ40, the Aβ42 isoform showed significantly higher levels in both the SAD and FAD groups compared to the ND group. In the FAD group, the Aβ42 levels in the *APPswe* mutant was, in stark contrast to the Aβ40 measures, lower than in the *PSEN1* mutants (**Fig. 3**).

In frontal cortex, in total, 20-fold higher levels of Aβ42 were obtained in SAD compared to ND (**Fig. 3g**), while in occipital cortex the increase was 10-fold (**Fig. 3h**). Compared to the ND group, both FAD and SAD groups showed significant elevation of Aβ42 levels in each of the frontal cortex fractions analyzed, (**Fig. 3a, 3c, 3e and 3g**). Furthermore, except for the TBS-soluble fraction (**Fig. 3a**), Aβ42 levels in the FAD cases were significantly higher compared to those of the SAD group in each frontal cortex fraction (**Fig. 3c, 3e and 3g**). This observation is in line with the fact that relatively more Aβ42 is produced by the mutants.

In all fractions of the occipital cortex, Aβ42 in both the FAD group and the SAD group was significantly higher than ND levels (**Fig. 3b, 3d, 3f and 3h**). Similar to frontal cortex, Aβ42 levels in occipital cortex were, except for the TBS-soluble fraction, (**Fig. 3b**) higher in the FAD group compared to the SAD group (**Fig. 3d, 3f and 3h**).

As expected, Aβ42 levels were higher in all the fractions analyzed from SAD and FAD brains, as compared to those from ND brain, hence, supporting a link between AD and the accumulation of neuropathogenic Aβ42 species in the brain.

### Analysis of Aβ43 Levels

Although Aβ43 is produced at lower levels than either Aβ40 or Aβ42, it is more prone to form neurotoxic aggregates [34] and thereby is potentially involved in AD pathogenesis. Consequently, we measured the Aβ43 levels in the fractions that were used for measuring Aβ40 and Aβ42.

Using frontal cortex, we found no significant differences in the TBS-soluble (**Fig. 4a**) Aβ43 levels between groups. Levels of Aβ43 in SDS and FA fractions were, as in the case of Aβ42, significantly higher in the FAD and SAD groups as compared to levels of the ND group (**Fig. 4c, 4e and 4g**).

In occipital cortex, the trend was similar to the results acquired from frontal cortex, with significantly higher levels in the FAD and SAD groups compared to the ND group in each SDS and FA fraction (**Fig. 4d, 4f and 4h**), while no significant differences were observed in the TBS-soluble fraction (**Fig. 4b**). Although the absolute Aβ43 levels were low compared to the abundant Aβ40 and Aβ42 isoforms (**Table S1**), the total Aβ43 mean concentration in frontal cortex was approximately 40 times higher than in the ND group (**Fig. 4g**); while for Aβ42, this difference between groups was approximately 20-fold (**Fig. 3g**). In contrast to Aβ42, no significant differences in Aβ43 levels were obtained between the SAD and FAD groups, suggesting that the studied mutations mainly affect Aβ42 production.

To summarize, Aβ43 is highly elevated in both SAD and FAD brain as compared to ND cases. However, in the most soluble fraction (TBS) a significant difference between cases was not obtained, which is in line with the strong aggregation propensity of Aβ43. The concentrations of Aβ in the samples from human brain utilized for this study differ largely between the Aβ40, Aβ42, and Aβ43 isoforms. The latter is found at low concentrations compared to Aβ40 and Aβ42 but is highly increased in the less soluble fractions obtained from SAD and FAD.

### Enrichment of Insoluble Aβ in Frontal and Occipital Cortex

**Figure 5:**
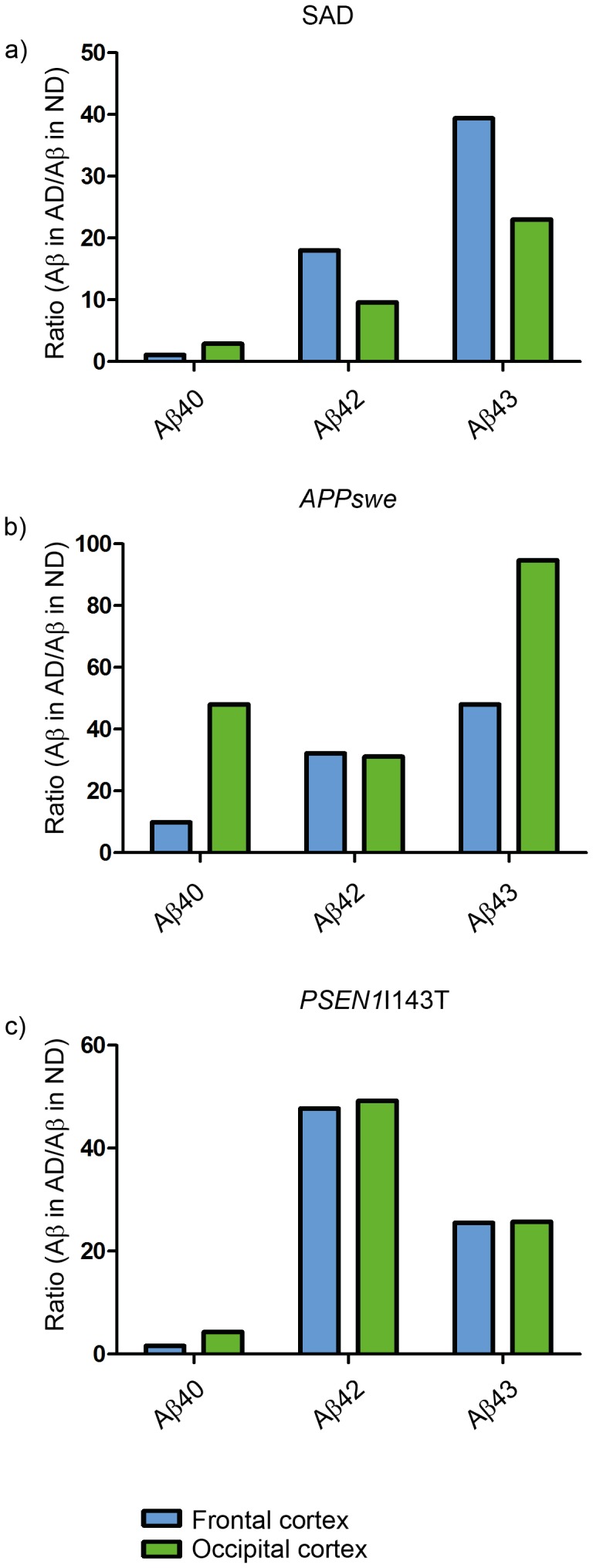
Scatter plots representing the enrichment of Aβ40, Aβ42 and Aβ43 in human brain. Absolute levels of Aβ in SDS-soluble and FA-soluble fractions were divided by levels in TBS-soluble fractions. ND: non-demented; SAD: sporadic Alzheimer disease; FAD: familial Alzheimer disease. **a**) Enrichment of insoluble Aβ in human frontal cortex; **b**) Enrichment of insoluble Aβ in human occipital cortex. *<0.05; **<0.01; #<0.05; ##<0.01.

The ratio between the insoluble fractions (SDS+FA) and TBS soluble (TBS) was calculated in order to analyze the potential enrichment of the different Aβ species in the less soluble fractions. We presumed that the more hydrophobic and, thereby, more aggregation prone peptides, Aβ42 and Aβ43, would be more enriched in the SAD and FAD groups than in the ND group. Indeed, Aβ42 and Aβ43 were similarly enriched in the SDS and FA fractions in both frontal and occipital cortex from SAD and FAD, while no such changes were observed for Aβ40 (**Fig. 5a and 5b**). These results are in line with the notion that Aβ40 is less prone to form neurotoxic aggregates than Aβ42 and Aβ43. The intra-individual differences were large in the SAD group, whereas most ND cases showed low enrichment of all the Aβ isoforms analyzed. In frontal cortex, we found that the enrichment of Aβ42 and Aβ43 was elevated in SAD compared to ND, and that Aβ43 was also increased in FAD compared to ND. In occipital cortex, Aβ42 enrichment was increased in both SAD and FAD compared to ND, while Aβ43 was increased only in the SAD group. The variation among cases may reflect disease severity, high enrichment indicating an advanced disease stage. In line with this theory, quantitative results from occipital cortex of both probable and possible AD cases included in the SAD group were in the lower range.

In Summary we Demonstrated that Aβ43 Accumulates Strongly in the SDS and FA Fractions, Similar to Aβ42.

### Ratios between Aβ Peptides

**Figure 6:**
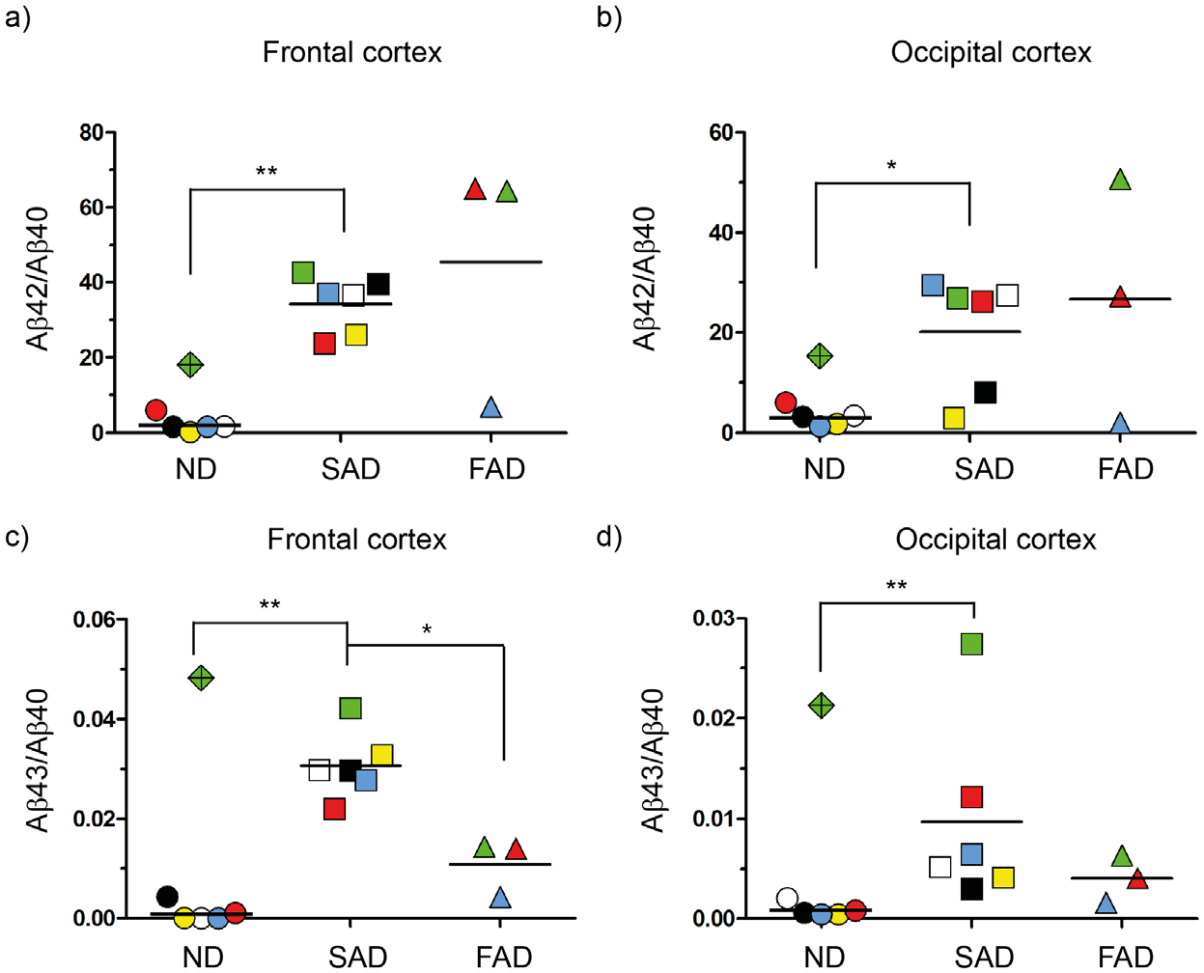
Aβ42/Aβ40 and Aβ43/Aβ40 ratios. a) Total Aβ42 level was divided by total Aβ40 level and by total Aβ43; b) Aβ43 total level was divided by total Aβ40 concentrations. *<0.05; **<0.01.

Calculating the ratios between Aβ isoforms is a popular way of presenting data about the different species. This is due to the fact that previous studies show the Aβ42/Aβ40 ratio is more highly correlated with AD than are the absolute levels of each Aβ isoform [36]. In order to explore the relation between Aβ40, Aβ42 and Aβ43, we calculated ratios between these peptides in all fractions from each individual case. The ratio between Aβ42 and Aβ40 was significantly greater in SAD compared to the ratios obtained for the ND group, in both frontal (**Fig. 6a**) and occipital cortex (**Fig. 6b**). Due to the high Aβ40 levels in the *APPswe* case the Aβ42/Aβ40 ratio was low. Consequently, the ratio for the FAD group was not significantly higher than for the ND group. However, both *PSEN1* mutation carriers had highly elevated Aβ42/Aβ40 ratios. The ratio between Aβ43 and Aβ40 in frontal cortex of the SAD group was significantly increased compared to ND, suggesting the importance of Aβ43 for AD pathology. On the other hand, the Aβ43/40 ratio in the FAD group was significantly lower than that for the SAD group (**Fig. 6c**). This is partly due to the extremely high Aβ40 levels identified in the *APPswe* case. Also in occipital cortex, the Aβ43/40 ratio of the SAD group was significantly more increased than that of the ND group (**Fig. 6d**). Meanwhile, there was no mean ratio difference to the FAD group.

In conclusion, our study supports findings of previous studies that the Aβ42/Aβ40 ratio is elevated in AD. Analyzing Aβ43 in the same fashion, we also demonstrated an increase in SAD. Hence, calculating Aβ43/Aβ40 side by side with Aβ42/Aβ40 may be an informative approach for demonstrating Aβ isoform differences between case groups.

## Discussion

In this paper, we performed a quantitative study of the levels of Aβ43 in fractions from human brain homogenates of different solubility, and compared those to the widely studied and more abundant Aβ40 and Aβ42 species.

Aβ43 was detected in all fractions of the SAD and FAD cases investigated in this study, whereas several of the ND cases displayed non-detectable Aβ43 levels and a few ND cases did not show any detectable Aβ42 level either. On the other hand, Aβ40 was present in all fractions of all cases. We speculate that the absence in several ND cases of the more neurotoxic Aβ42, and especially Aβ43, is related to the fact that these subjects were neurologically healthy at death. Yet, one of the ND cases (green diamond) exhibited very high levels of Aβ43 in the detergent soluble fraction (**Fig. 4c** and **Fig. 4d**) and relatively high levels of Aβ43 in the formic acid fraction of frontal cortex (**Fig. 4e**). This case also had high Aβ40 and Aβ42 levels in the soluble fractions of the frontal cortex (**Fig. 2a**, **Fig. 3a**). We hypothesize that this particular individual was in the pre-symptomatic phase of AD, demonstrated by early Aβ43 accumulation. This individual had not been diagnosed with a dementing illness before death but the neuropathological examination revealed a few diffuse and senile plaques in both hippocampal and neocortical tissue. Due to the risk that this case was a pre-symptomatic case of AD, we decided to exclude the case from all the grouped analysis of this study, but to keep the case as a unique symbol in the scatter plots.

The two amino acids that differentiate Aβ42 from Aβ40 are isoleucine and alanine, the former of which is hydrophobic and has a high β-sheet propensity, leaving Aβ42 susceptible to aggregate [17]. Aβ43 ends with the additional amino acid, threonine, which has among the highest propensity of all amino acids to form β-sheet structures [42]. Based on its primary sequence, it is reasonable to predict that Aβ43 is more prone to aggregate than Aβ42. We and others have previously shown, that Aβ43 is slightly more hydrophobic than Aβ42 and shares a similar ability to aggregate as does Aβ42 [17], [27]. Saito and colleagues also demonstrated that the additional threonine residue of Aβ43 seems to strengthen the β-sheet propensity of the Aβ peptide as well as its neurotoxic effect [34]. These authors further demonstrated that Aβ43 can drive Aβ42 polymerization. Together, these studies show that Aβ43 has features that promote peptide aggregation. Hence, we suggest that both Aβ42 and Aβ43 are important players in AD plaque formation. Consequently, we show that Aβ42 and Aβ43, but not Aβ40, are enriched in the less soluble fractions from both frontal and occipital cortex of SAD cases (**Fig. 5a and 5b**).

Still, the absolute levels of Aβ43 in human brain are low, compared to Aβ40 and Aβ42 (**Fig. 2**, **3** and **4**). However, since Aβ43 is shown to be more neurotoxic than both Aβ40 and Aβ42 [34], low levels may still have large impact. In a previous study from our group, mass spectrometry of plaque cores revealed that Aβ43, but not Aβ40, was present in most of the cases studied [27]. In the same study, we showed that Aβ43 also was present in the SDS-insoluble fractions (corresponding to the FA-fractions analyzed in the present study), where high levels for both Aβ40 and Aβ42 were detected. An augmented relative level of Aβ43 in the fractions containing plaque cores, compared to the more soluble fractions analyzed in the present paper, would argue for a seeding effect of Aβ43, suggesting Aβ43 is a suitable candidate to indicate an early neurodegenerative process in the AD brain. Actually, a recent study showed that a decreased level of Aβ43 could be observed in CSF from MCI and AD [37].

The presence of amyloid deposits in vessels, cerebral amyloid angiopathy (CAA) are common features in aging and in AD, occurring in 10–40% of all elderly and in 80–100% of AD patients [43], [44]. A uniform topographical distribution has not been determined for CAA, but such deposits predominate in the occipital lobe [45] and are mainly composed of Aβ40. We noticed very different Aβ40 amounts in the TBS soluble fractions obtained from occipital versus frontal cortex. Such discrepancy between regions may possibly be associated with CAA. Accordingly, comparing the occipital and frontal cortex regions, we found that the occipital region had approximately 10-fold higher levels of Aβ40 in the ND group and 30-fold higher levels in the SAD and FAD groups (**Fig. 2a and 2b**). This difference was less pronounced in the total Aβ40 amounts, where there was a 2- or 4- fold difference, respectively (**Fig. 2g and 2h** **)**. In line with this notion, we also observed almost a 2-fold increase of the Aβ42/40 ratio in frontal compared to occipital cortex (**Fig. 6a and 6b**). Further, Aβ43/40 ratios were 3-fold higher in SAD frontal cortex compared to occipital cortex (**Fig. 6c and 6d**).

We, here, analyzed cortical Aβ content from two individuals from the same family carrying the *PSEN1* I143T mutation (**Fig. 2** **,** **3** and **4**). The Aβ43 levels in these FAD cases were in the lower range of concentrations found in the SAD cohort; however, these differentiate from the ND cases, in which all but one case consistently presented values close to the detection level. Quantification of Aβ in the *PSEN1* I143T mutation affected individuals revealed an increased Aβ42/40 ratio (**Fig. 6a, 6b**), but not increased Aβ43/40 ratio (**Fig. 6c, 6d**), which would argue for a mutation induced peptide pathway selectivity (48→45→42→38). Previous studies on *PSEN1* and Aβ43 are limited in number. However, in one study on mouse embryonic fibroblasts derived from PS1R278I homozygotes, Aβ43 levels were increased concomitantly with decreased Aβ40 levels, which was explained by a proposed mechanism on deficit in conversion from Aβ43 to Aβ40 [34]. However, our data does not support this proposed mechanism, which may be due to the fact that we study another *PSEN* mutation or, alternatively, may reflect differences between mouse and human brain. We also analyzed one case carrying the APP_Swe_ mutation. This case showed extremely high Aβ40, high Aβ43 levels and moderate Aβ42 levels (**Fig. 2** **,** **3** and **4**). These results are in line with the previously suggested preference for the 49→46→43→40 peptide pathway generated through APP cleavage [12].

In summary, we have quantified Aβ43 in two cortical regions from SAD and FAD human brains, prepared according to different solubility. Aβ43 was detectable in all AD fractions analyzed and enriched in the less soluble fractions in a manner similar to Aβ42. We suggest that Aβ43 is important for AD, where it may play an initiating role in Aβ amyloid plaque formation.

## Supporting Information

Table S1 **Absolute levels of Aβ40, Aβ42 and Aβ43 in frontal and occipital cortex.** TBS fraction concentrations are in pmol/g protein and SDS, FA and total fractions are expressed in nmol/g protein.
(TIF)
