# Correction: The Pathogenic Aβ43 Is Enriched in Familial and Sporadic Alzheimer Disease

**DOI:** 10.1371/annotation/9e7b419c-4f85-4922-89f6-b7e4d30149c4

**Published:** 2013-05-16

**Authors:** Anna Sandebring, Hedvig Welander, Bengt Winblad, Caroline Graff, Lars O. Tjernberg

There were numerous errors in the text of the article. Correct versions of this text can be found here: http://www.plosone.org/corrections/pone.0055847.001.cn.tif

