# Correction: The Pathogenic Aβ43 Is Enriched in Familial and Sporadic Alzheimer Disease

**DOI:** 10.1371/annotation/b4d7aee1-6578-4e73-b15d-67aef9b3da8c

**Published:** 2013-05-23

**Authors:** Anna Sandebring, Hedvig Welander, Bengt Winblad, Caroline Graff, Lars O. Tjernberg

The cause of the errors in the Correction notice was the use of the incorrect manuscript during the typesetting process of the article by PLOS. 

