# Supplementary figures and images for: The Pathogenic Aβ43 Is Enriched in Familial and Sporadic Alzheimer Disease
Source: PLoS One. 2013 Feb 11;8(2):e55847. doi: 10.1371/journal.pone.0055847 (PMC3569467; doi:10.1371/journal.pone.0055847)

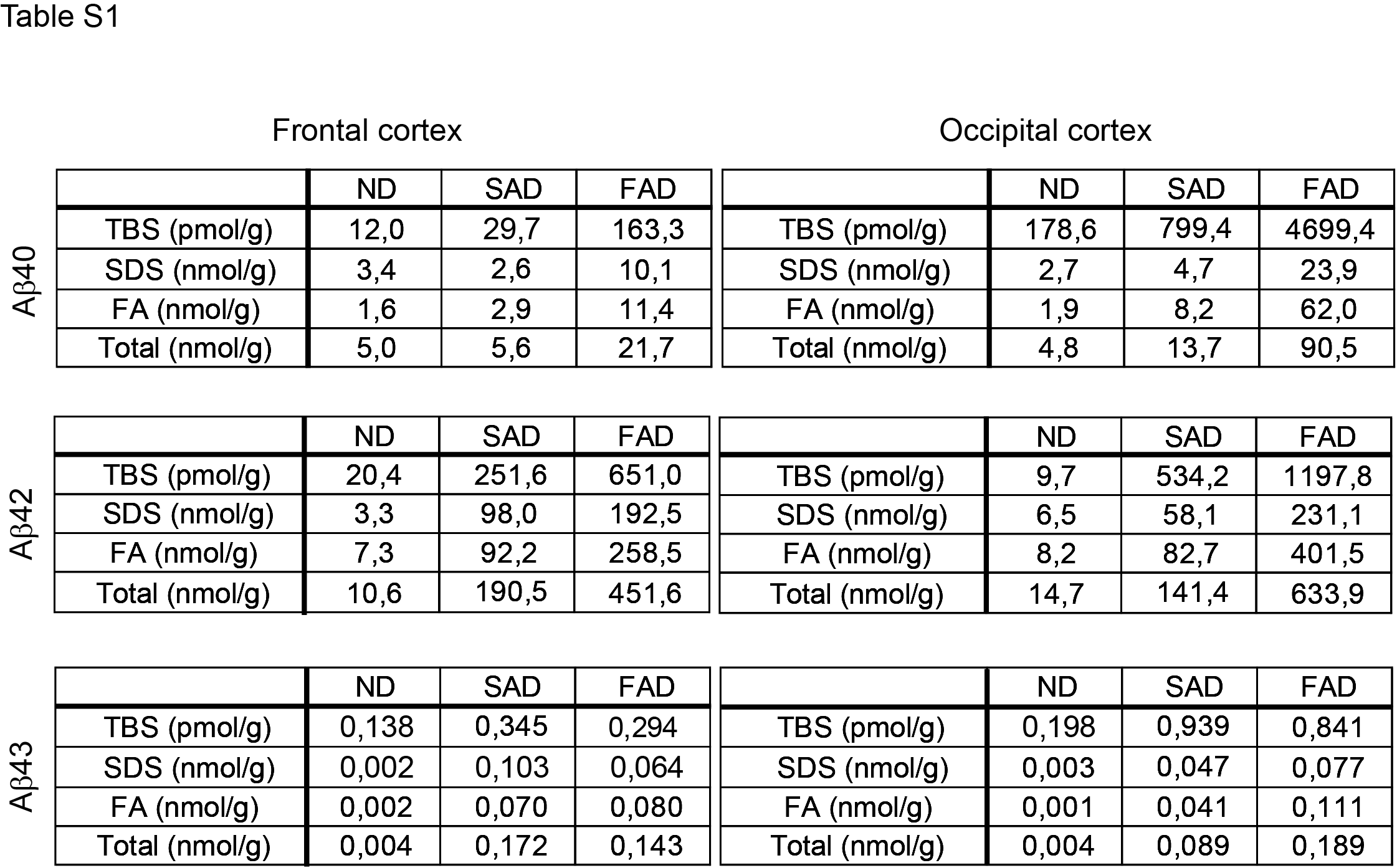

Supplement: Table S1 — Absolute levels of Aβ40, Aβ42 and Aβ43 in frontal and occipital cortex. TBS fraction concentrations are in pmol/g protein and SDS, FA and total fractions are expressed in nmol/g protein. (TIF) [file pone.0055847.s001.tif]
